# Khat chewing in pregnant women associated with prelabor rupture of membranes, evidence from eastern Ethiopia

**DOI:** 10.11604/pamj.2020.36.1.22528

**Published:** 2020-05-01

**Authors:** Tesfaye Assebe Yadeta, Gudina Egata, Berhanu Seyoum, Dadi Marami

**Affiliations:** 1 School of Nursing and Midwifery, College of Health and Medical Sciences, Haramaya University, Harar, Ethiopia; 2 School of Public Health, College of Health and Medical Sciences, Addis Ababa University, Addis Ababa, Ethiopia; 3 Armauer Hansen Research Institute, Addis Ababa, Ethiopia; 4 Department of Medical Laboratory Sciences, College of Health and Medical Sciences, Haramaya University, Harar, Ethiopia

**Keywords:** PROM, Khat plant, Catha edulis, khat chewing, pregnancy, Harar, Ethiopia

## Abstract

**Introduction:**

Prelabor rupture of membranes (PROM) is a major factor that affects pregnancy outcome. Results from previous studies have suggested that there is an association between pregnant women, khat chewing and preterm birth, but evidence of association with PROM is sparse. This study therefore aims at identifying association between khat chewing in pregnancy and premature rupture of membranes in eastern Ethiopia.

**Methods:**

A health facility-based cross-sectional study was conducted among 1688 pregnant women who came for delivery service in Harar town, Eastern Ethiopia between June to October in 2016. Data were collected using a pre-tested structured questionnaire and checklist to extract data from the medical record. The association between khat and PROM was examined using logistic regression analysis. A statistical significance was declared at p-value < 0.05.

**Results:**

Of the 1688 pregnant women who participated in the study, 397 had prelabor rupture of the membranes, representing a proportion of 23.5% [(95% CI: (21.5, 25.6%)]. Of these 397 prelabor rupture of the membranes 198 (31.53%) were from Khat chewing mothers and, 199(18.77%) were from non-khat chewing mothers. After controlling for potential confounders, the multivariable logistic regression analysis revealed the odds of PROM was 1.51 times higher among khat chewed pregnant women [AOR = 1.51; 95% CI; (1.11, 2.07)] were had no khat chewing.

**Conclusion:**

This study found a significant association between khat chewing in pregnancy and PROM. Efforts to reduce PROM need to consider prevention of khat chewing in pregnancy. A specific strategy need to protect pregnant women from khat chewing.

## Introduction

Prelabor rupture of membranes (PROM) is rupture of membranes that occurs before the onset of labor. Membranes rupture before labor and before 37 weeks of gestation is referred to as preterm PROM. Term Prelabor Rupture of Membranes (term PROM) is defined as rupture of the membranes prior to the onset of labor at or beyond 37 weeks gestation [1]. Prelabor rupture of membranes complicates approximately 8% of pregnancies. Prelabor rupture of membranes has been shown to increase the risk of cord prolapse, cord compression and placental abruption, maternal chorioamnionitis, infectious endometritis, neonatal sepsis, and perinatal death. Preterm PROM is responsible for 1 of every 3 preterm births and is associated with significant morbidity for both women and neonate [2-7]. Widespread khat chewing in pregnancy, in east African countries, including Ethiopia is recognized as an important public health concern [8,9]. Khat is chewed for its central nervous system stimulant properties. The major pharmacologically active constituent of the fresh khat leaf is cathinone which is extracted by mastication [10]. Khat chewing in pregnancy women were at risk for preterm labor, labor induction and fetal distress, intra uterine fetal death (IUFD), lower mean hemoglobin concentration at delivery, low birth weight, higher perinatal mortality and congenital malformations [11]. During khat chewing, the leaves are simply kept in the side of the cheek to masticate over time. Prolonged mastication of khat affects oral hygiene status of chewers. The incidence of gingival bleeding, inflammation, ulcers on the oral mucosa, and gingival recession was significantly higher in khat-chewers [12]. Khat use increase the risk of esophageal cancer [13]. Periodontal disease is associated with PROM [14,15]. Exposure to contaminated sources during khat chewing was reported to cause bacteremia and meningitis [16]. Khat chewing grown with chemical pesticides may cause considerable adverse health effects in khat users [17]. Khat may be a contributing factor in the pathological diseases found among khat users like liver disease, coronary problems, cancers of the digestive tract and incidents of domestic violence [18,19]. Despite impact of khat on adverse pregnancy outcome, pregnant women in the study area were khat chewing. This may be due to lack of information on the health impact of khat during pregnancy. The aim of this study was to perform an investigation on the association of khat chewing in pregnancy and PROM among pregnant women.

## Methods

**Study setting:** this study was performed in three health facilities (Hiwot Fana Specialized University Hospital, Jugal Hospital and Arategna Health Center) found in Harar town, Eastern Ethiopia. Harar is located 510 km east of the capital city of Ethiopia, Addis Ababa. Ethiopia is among widely distributed khat chewing country of east Africa [8,20].

**Study design and sampling:** a cross-sectional study was done between June and October 2016. The study participants were 1688 singleton pregnant women who came for delivery services at labour and delivery rooms of the selected health facilities. Study participants were taken proportional to the client load of the selected health facilities.

**Data collection:** data were collected using a pretested and structured questionnaire. In addition, a checklist was used to extract data from the medical record. The data collection tools were developed by reviewing various previous related data collection instruments and articles [1,21-23]. The questionnaire included socio-demographic characteristics of the participant like (age of mothers, residence of mothers, educational status, and khat chewing in pregnancy), obstetric and gynecologic history (history of abortion, history of preterm, history of PROM,), current pregnancy obstetric and gynecologic situation (ANC, parity and anemia). The original questionnaire was prepared in English language and later translated into the local languages (Amharic and Afan Oromo) for data collection. Forward and backward translations were performed by language experts. A six-day training was given to attending midwives and the supervisors who worked as data collectors. The training for data collectors and supervisors addressed issues related to data collection procedures and tools, interviewing techniques, medical record review procedures. A research manual was prepared to guide training, data collection and management.

**Measurement:** prelabor rupture of membranes (PROM) is rupture of membranes that occurs before the onset of labor. Membranes rupture before labor and before 37 weeks of gestation is referred to as preterm PROM. Term Prelabour Rupture of Membranes (term PROM) is defined as rupture of the membranes prior to the onset of labour at or beyond 37 weeks gestation [1]. Premature rupture of membranes was the dependent variable in this study and this information was obtained from medical records. It was labelled as “yes” and “no” with a code of 1 and 0, respectively. Khat chewing in pregnancy, age of mothers, residence of mothers, educational status, history of abortion, history of preterm, history of PROM, ANC, parity and anemia were independent variables. Khat chewing in pregnancy was collected based on maternal replay for the question “have you chewed khat during current pregnancy?”, and labeled as “yes” and “no” and coded with 1 and 0, respectively. Age of the mother was documented based on maternal reply and later grouped as 15-19 and 20+ with code 1 and 2, respectively for analysis. Maternal education was grouped as “literate” for those who could read and write and all others as “illiterate” and was coded 0 and 1, respectively. Antenatal care visit was obtained from maternal reply and labeled as “yes” and “no” and coded with 1 and 0, respectively. Number of births (parity) were grouped as one primiparity (one delivery), multiparous (2-4 delivery) and grand multiparous (= 5 deliveries) and code with 1, 2 and 3, respectively [20]. History of abortion, history of preterm, and history of PROM was documented based on maternal reply and labeled as “yes” and “no” and coded with 1 and 0, respectively.

**Statistical analysis:** data were double entered and cleaned using Epi-Data version 3.1 and analyzed using STATA version 14. Summary and proportions of the variables were computed against PROM. The logistic regression model was used to assess the association between independent and the outcome variable (PROM). Multicollinearity was tested using the Variance Inflation Factor (VIF) and the tolerance test. Correlation matrix and covariate matrix were tested for the final model. The Hosmer-Lemeshow goodness-of-fit tests were used to test for model fitness [24]. Bivariate analysis was first made and the variables with a p-value less than 0.25 were considered for multivariable analysis, along with maternal age, important predictors of PROM, regardless of the cut-off point for p value. The association between khat chewing in pregnancy and the outcome variable (PROM) was analyzed by controlling for other potential confounding variables, a p value of less than 0.25 in bivariate analysis and maternal age. Adjusted Odds Ratio (AOR) along with 95% confidence interval was estimated to assess the strength of the association. Statistical significance was declared at a p-value < 0.05.

**Ethical approval and consent to participate:** the study material was reviewed and approved by the Institutional Health Research Ethics Review Committee of the College of Health and Medical Sciences at Haramaya University. Permission was obtained from each of the health facilities involved in the study. Written consent was obtained from each woman before commencement of data collection. Women experienced PROM was treated. Neonates with health problems were treated and immediate referral was also facilitated.

## Results

Of the 1688 pregnant women who participated in the study, 397 had prelabor rupture of the membranes, representing a proportion of 23.5% [95% CI:(21.57,7 25.6%)7]. Of these 397 prelabor rupture of the membranes 198(31.53%) were from mother had khat chewing and, 199(18.77%) were from non-khat chewing (Table 1). Bivariable analysis revealed, khat, educational status, history of abortion, history of preterm, history of PROM, ANC, parity, and anemia were found to have a significant association with PROM (Table 1).

**Table 1:** Basic characteristics of study subjects by premature rupture of the membranes and unadjusted odds ratio, Harar town, Eastern Ethiopia, 2016

Characteristics		Total n=1688	Experience PROM		Crude OR CI			P value
			No (%)	Yes (%)				
**Khat**	No	1,060	81.23	18.77	1			
	Yes	628	68.47	31.53	1.99	1.58	2.50	0.000
**Age of mothers**	≤19	301	75.08	24.92	1			
	20 and above	1,387	76.78	23.22	0.911	0.68	1.21	0.528
**Residence of mothers**	Urban	943	75.19	24.81	1			
	Rural	745	78.12	21.88	0.84	0.67	1.06	0.158
**Educational status**	Literate	894	78.52	21.48	1			
	Illiterate	794	74.18	25.82	1.27	1.01	1.59	0.036
**History of abortion**	No	1,388	78.82	21.18	1			
	Yes	300	65.67	34.33	1.94	1.48	2.55	0.000
**History of preterm**	No	1,657	77.07	22.93	1			
	Yes	31	45.16	54.84	1.62	1.21	2.17	0.001
**History of PROM**	No	1,456	81.66	18.34	1			
	yes	232	43.97	56.03	3.38	1.64	6.97	0.001
**ANC**	No	561	70.23	29.77	1			
	Yes	1,127	79.59	20.41	0.60	0.47	0.76	0.000
**Parity**	1	722	81.72	18.28	1			
	2-4	755	74.30	25.70	1.54	1.20	1.98	0.001
	≥5	211	66.35	33.65	2.26	1.60	3.19	0.000
**Anemia**	No	1,535	79.54	20.46	1			
	Yes	153	45.75	54.25	4.61	3.27	6.48	0.000

**Multicollinearity test:** multicollinearity was tested using the Variance Inflation Factor (VIF) and tolerance test. VIF for all variables was less than 10, and tolerance test was greater than 0.2. This indicate there was no multicollinearity problem [25] (Table 2).

**Table 2:** Multicollinearity test output among predictor variables, Harar, Eastern Ethiopia, 2016

Variable	Variance inflation test (VIF)	Tolerance test (1/VIF)
Khat chewing in pregnancy	1.07	0.930794
Age of mother	1.06	0.944123
Residence of mothers	1.36	0.734311
Maternal education	1.44	0.695489
History of abortion	1.15	0.869117
History of preterm delivery	1.05	0.949141
History of PROM	1.18	0.849108
Antenatal care follow up	1.25	0.801577
Parity	3.40	0.293837
	3.64	0.274827
Anemia in mother	1.08	0.925358
Mean	1.61	

**The multivariable logistic regression analysis:** the multivariable logistic regression analysis revealed the odds of PROM was 1.51 times higher among khat chewed pregnant women [AOR = 1.51; 95% CI; (1.11,2.07)] who had no khat chewing. In addition, the odds for pregnant women who had history of PROM [AOR = 4.77; 95% CI; (3.35,6.79)], and mother experience anemia [AOR = 3.18; 95% CI; (2.09,4.85)] had increased odds of experiencing PROM. Age twenty and above year 51% [AOR =0.49; 95% CI; (0.27, 0.87)], rural resident 44% [AOR =0.56; 95% CI; (0.39, 0.80)], and attended ANC follow-up 37% [AOR =0.63; 95% CI; (0.44,0.89)] had lower odds of experiencing PROM (Table 3).

**Table 3:** Multivariable output of factors associated with PROM among pregnant women, Harar, Eastern Ethiopia, 2016

Characteristics		AOR	95% Conf. Interval		P value
**Khat chewing in pregnancy**	No	1			
	Yes	1.51	1.11	2.07*	0.009
**Age of mother**	≤19	1			
	20 and above	0.49	0.27	0.87*	0.017
**Residence of mothers**	Urban	1			
	Rural	0.56	0.39	0.80	0.002
**Maternal education**	Literate	1			
	Illiterate	1.04	0.72	1.51	0.813
**History of abortion**	No	1			
	Yes	1.22	0.86	1.73	0.244
**History of preterm delivery**	No	1			
	Yes	1.83	0.80	4.15	0.146
**History of PROM**	No	1			
	yes	4.77	3.35	6.79*	0.000
**Antenatal care follow up**	No				
	Yes	0.63	0.44	0.89*	0.01
**Parity**	1	1			
	2-4	1.22	0.64	2.34	0.531
	≥5	1.01	0.48	2.13	0.962
**Anemia in mother**	No	1			
	Yes	3.18	2.09	4.85*	0.000

AOR: Adjusted Odds Ratio

## Discussion

This study showed an association between PROM and khat chewing in pregnancy, after controlling for other potential confounders. Similar findings were reported from Yemen [11]. Khat is consumed by plucking off the leaves of the tree and rolling them into little balls that are pushed into the side of the cheek, where they form a large bolus (soft mass of leaves). The practice is usually referred to as khat chewing, but in fact the leaves are simply kept in the side of the cheek to masticate over time. Prolonged mastication of khat affects oral hygiene status of chewers. The incidence of ulcers on the oral mucosa, gingival bleeding, gingival recession is high [12]. Khat use increase the risk of esophageal cancer [13]. Periodontal disease is associated with PROM [14,15]. The link between periodontal disease and preterm delivery is a possible translocation of periopathogenic bacteria to the placenta and amniotic fluid as well as a systemic response to this chronic inflammatory disease [25,26]. Other studies have reported, khat chewing pregnant women were at risk for infection, due to exposure to contaminated sources during chewing [16]. Infection in the female reproductive tract can cause prelabor rupture of the membranes and induce premature labor [5]. Prolonged PROM significantly increases the risk of placental infection [6]. Histological chorioamnionitis (HCA) and funisitis increase the risk of adverse perinatal outcome in PPROM pregnancies [7]. Preterm prelabor rupture of membranes is associated with Intra-amniotic infection and perinatal deaths [2]. The implication of khat chewing grown with chemical pesticides may cause considerable adverse health effects in khat users need further investigation [17]. At term, programmed cell death and activation of catabolic enzymes, such as collagenase and mechanical forces, result in ruptured membranes [8]. The aetiology of PROM has been shown to be multifactorial, and increasing evidence has regarded exposure to environmental pollutants as risk factors for PROM like lead exposure [9,27].

Experiencing anemia was one of the predictors´ of PROM. Similar findings were reported [11,28]. Khat chewing results in lowering mean hemoglobin concentration at delivery [11]. Maternal anemia results in hypoxic conditions of the feto-placental unit and thus is a suitable model to study the effects of hypoxia on villous proliferation in human placentae [29]. Additionally, in this study pregnant woman who had history of PROM was an increase the risk of PROM. Similar findings were reported in other studies conducted in different part of the world [30-32]. The cause of PROM is obscure, however the reason may be due to maternal genital bacterial colonization, maternal medical and obstetric complications that are predictors for PROM, there may be recurrence, in the study area where there is no screening [33]. Women presenting with premature rupture of the membranes, intrauterine infection, and oligohydramnios are at risk of placental abruption [34]. Aspirin and progesterone may be used in preventing and delaying preterm birth recurrence [35,36]. In this study, antenatal care follow up was one of the predictors. Similar findings were reported in Pakistan [37]. The reason may be treatment was given when the pregnant women come to health center with problems. Despite this, ANC follow up was a protective for PROM. Only 32% of pregnant women had four recommended ANC visits in Ethiopia [15]. To achieve the sustainable development goal to end all preventable maternal and perinatal mortality and morbidity in relation with PROM, health promotion programs must better target those communities where maternal health care utilization is low. Effective interventions are therefore needed to improve the ANC coverage as part of a PROM reduction strategy. Age twenty and above and being a rural resident were reduced PROM recurrence. This needs further investigation. There are a number of initiatives that are currently being worked on improving maternal and newborn health care delivery in Ethiopia. The Health Extension Program, launched by the Federal Ministry of Health of Ethiopia in 2003, involves the placement of Health Extension Workers (HEW) in rural villages to deliver mostly disease prevention and health promotion activities. There is an increase in the reporting of sick children, access to ANC and post-natal care [38]. Despite these successes, khat chewing in pregnancy is spreading very fast. The community may have no understanding on its associated risk. Our study findings reveal that there is still much work to be done at the community level to protect pregnant women from the adverse effect of khat chewing.

**Strengths and limitations of this study:** this study was conducted with a large sample size which allowed us to adjust for other potential risk factors for prelabor rupture of membranes. Although many potential confounders were taken into account for analysis, other confounding factors may remain. Clinical assessment and periodontal status was not done.

**Funding:** this study was financially supported by Haramaya University. The funding body did not play any role in the design of the study, collection, analysis, interpretation of data, and in writing the manuscript.

## Conclusion

This study demonstrated a significant association between khat chewing in pregnancy and PROM. Efforts to reduce PROM need to consider prevention of khat chewing in pregnancy. Policy and programmatic efforts to optimize pregnancy outcome should consider khat use as an important determinant.

### What is known about this topic

- Khat chewing affects oral hygiene status of chewers;
- Periodontal disease is associated with prelabor rupture of membranes;
- Prelabor rupture of the membranes increase the risk of infection.

### What this study adds

- Possible translocation of periopathogenic bacteria to the placenta and amniotic fluid as well as a systemic response to this chronic inflammatory disease;
- This study rises the discussion around the association between khat chewing in pregnancy and prelabor rupture of the membranes.

## Competing interests

The authors declare no competing interests.
